# Awareness, perception and utilization of a mobile health clinic by people who use drugs

**DOI:** 10.1080/07853890.2021.2022188

**Published:** 2022-01-04

**Authors:** Suzanne M. Grieb, Robert Harris, Amanda Rosecrans, Katie Zook, Susan G. Sherman, Adena Greenbaum, Gregory M. Lucas, Kathleen R. Page

**Affiliations:** a Department of Pediatrics, Center for Child and Community Health Research, Johns Hopkins University School of Medicine, Baltimore, MD, USA; b Baltimore City Health Department, Baltimore, MD, USA; c Division of Infectious Diseases, Johns Hopkins University School of Medicine, Baltimore, MD, USA; d Department of Health, Behavior and Society, Johns Hopkins Bloomberg School of Public Health, Baltimore, MD, USA

**Keywords:** Mobile health care, people who use drugs, opioid use disorder, low-barrier buprenorphine, drug use stigma

## Abstract

**Introduction:**

People who use drugs (PWUD) face a multitude of barriers to accessing healthcare and other services. Mobile health clinics (MHC) are an innovative, cost-effective health care delivery approach that increases healthcare access to vulnerable populations and medically underserved areas. There is limited understanding, however, of how PWUD perceive and experience MHCs.

**Methods:**

Semi-structured interviews were conducted with 31 PWUD – 16 who had received care (clients) on an MHC (The Spot) and 15 who had not (non-clients) – to explore their perceptions and utilization of an MHC partnered with a mobile syringe services program in Baltimore, Maryland. Data analysis of the text was conducted using an iterative thematic constant comparison process informed by grounded theory.

**Results:**

Clients and non-clients, once aware of the MHC, had positive perceptions of The Spot and its benefits for their individual health as well as for the wellbeing of their community. These sentiments among clients were largely driven by access to low-barrier buprenorphine and service delivery without stigma around drug use. However, lack of general awareness of the spot and specific service offering were barriers to its use among non-clients.

**Discussion:**

MHCs provide an important opportunity to engage PWUD in healthcare and to expand buprenorphine use; however, even with accessibility near where PWUD access injection equipment, barriers to its use remain. Peer dissemination may be able to facilitate program information sharing and recruitment.

**KEY MESSAGES**
- People who use drugs perceive a mobile health clinic in their neighbourhood as a benefit to their communities and themselves by improving access to healthcare services, providing access to low-threshold buprenorphine dispensation, and offering services without drug use stigma.
- People who use drugs learned about a mobile health clinic in their neighbourhood largely through word-of-mouth. As a result, people received limited information about the mobile health clinic services creating a barrier to its use.

## Introduction

The U.S. is in the midst of an opioid epidemic with continued increases in opioid-related morbidity and mortality that were worsened by the COVID-19 pandemic [1–4]. People who use drugs (PWUD) experience acute complications of overdose and injection-related skin and soft tissue infections (i.e. abscesses, cellulitis), and, compared to the general population, are more likely to have a higher burden of chronic comorbid medical conditions, including mental health conditions [5–9]. Importantly, polysubstance use is now common among persons who use opioids, and the U.S. is now experiencing a “fourth wave” of the opioid overdose crises [10]. Polysubstance use contributes to increased morbidity and mortality [11–15] due to pharmacokinetic or pharmacodynamic interactions [12] and/or increased behavioural risk behaviours such as more frequent injecting and syringe sharing [16,17].

PWUD, however, face a complex array of barriers to accessing medical care. These barriers occur at the individual – (e.g. low healthcare prioritization), interpersonal – (e.g. drug-use stigma among providers), healthcare systems – (e.g. difficulty navigating healthcare systems, lack of identification, the burden of appointment), and structural – (e.g. homelessness and housing instability, lack of insurance) level [18–22]. As a result, PWUD has low primary care utilization [23–25], delays seeking medical care [22,26,27], and more frequently utilizes emergency medical care than the general population [28–31]. While these barriers exist for other marginalized populations, the high prevalence and oftentimes co-occurrence results in a complexity that creates a need for tailored efforts to engage them in healthcare. The impacts of these barriers are amplified in the context of illegality [21,22,32].

Many of the multilevel barriers to accessing healthcare also impede access to medications for opioid use disorder (MOUD). MOUD, specifically methadone or buprenorphine, is the most effective treatment for opioid use disorder (OUD) and has been shown to reduce opioid use and risk of overdose [32,33], increase treatment retention [34,35], decrease use of emergency medical care [36], decrease transmission of HIV and hepatitis [37–39], and improve quality of life and overall health status [33,34]. Patients utilizing methadone must receive their medication at opioid treatment programs (OTPs), which are closely regulated at the federal and state level. Buprenorphine can be accessed through OTPs or from a qualified provider [40,41]. Providers, however, are limited in the number of patients they may treat with buprenorphine, and numerous provider barriers exist that contribute to the limited number of providers seeking qualification and the underuse of buprenorphine among those qualified (e.g. regulatory hurdles, lack of time and clinic resources, lack of available mental health or psychosocial support services) [41–44]. These federal regulations and the structural barriers they create contribute to the significant gaps observed between treatment needs and capacity [45,46]. This service gap remains a critical barrier to health equity for this population in need of accessible and tailored services.

People with OUD additionally face barriers to accessing MOUD beyond the service gap. Program and systems-level barriers such as requirements for admission into an OTP and compliance requirements can be burdensome for patients [42,47–49]. Structural factors, such as lack of insurance and unstable housing, may limit patients from accessing and/or adhering to their treatment [50]. Furthermore, OUD and MOUD are highly stigmatized, even among health professionals. People utilizing MOUD experience a layering of stigma – they are stigmatized because of their condition (OUD) as well as for their treatment intervention (MOUD) [30,51]. This stigma can result in healthcare and treatment delays or avoidance by PWUD [22,51–55].

Given the challenges PWUD experiences in accessing healthcare services and MOUD, low-threshold service models have the potential to improve utilization. Low-threshold service models emphasize engagement and harm reduction approaches while aiming to reduce barriers to access [55–57]. Mobile health clinics (MHC) are an innovative, cost-effective health care delivery approach that can provide low-threshold services, and are increasingly used to provide healthcare to vulnerable populations and medically underserved areas [58,59]. MHC provides a community-centred approach to healthcare delivery that seeks to address geographical, structural, and social barriers to healthcare access [60,61]. They can provide a range of health services, including, but not limited to, primary care and preventative care, mental health services, chronic disease management, substance use treatment, and urgent care, with nearly half of MHC providing more than one type of service. Additionally, MHC often provides assistance with social services [58,62].

MHC remain underutilized, however, and Yu et al. have identified four categories of challenges and potential limitations to implementing MHC – risk of fragmented care, financial challenges, space and clinic structure barriers, and challenges in logistical planning [61]. For example, because many MHC is not fully incorporated into a healthcare system, continuity of care for MHC patients can be difficult. Due to the small space, the availability of specific services and/or quality of certain services may be impacted as all machines must be portable [63]. Patient confidentiality may also be difficult to maintain in this small space, requiring MHC to develop creative strategies to work around the spatial limitations to privacy [64,65]. Some MHC have also reported challenges for staff recruitment and retention as they need culturally competent staff willing to work in the small space in underserved communities.

Despite these challenges, MHC are a promising tool to provide healthcare to vulnerable populations and medically underserved areas [58,59], including people who use drugs. Work by Altice and colleagues has shown that MHC are acceptable, feasible, and effective for serving people who use drugs, including for vaccination, screening and treatment of latent tuberculosis, HCV screening, and treatment facilitation for persons living with HIV [66–69]. A qualitative exploration of MHC user’s perceptions of and experiences with an MHC has been conducted with various vulnerable and/or medically marginalized groups [70,71]; however, despite the importance of this understanding for providing optimal care [72–74], little has considered those of PWUD, who face unique barriers to healthcare. Accordingly, the objective of this paper was to explore the perceived benefits and barriers of utilizing an MHC co-located with a mobile syringe services program by PWUD in Baltimore, Maryland.

## Methods

### Healthcare on the spot

Healthcare on the Spot (henceforth referred to as The Spot), an MHC jointly operated by the Baltimore City Health Department (BCHD) and the Johns Hopkins School of Medicine, was initiated in 2018 to increase access to healthcare among people who use drugs [75]. Like much of the U.S., Baltimore, Maryland is experiencing an opioid crisis. Opioid-related overdose mortality increased 140% from 354 in 2015 to 851 in 2019; 95% of this mortality in 2019 was due to fentanyl [76]. The Spot provides clinical care in five specific sites selected from established BCHD’s mobile Syringe Services Program (SSP) sites; each site has high drug traffic and use. At some locations, the two vans are parked next to each other. At other sites, however, the vans are separated by time or space depending on the logistics and the surrounding community’s request. The Spot van includes two patient exam rooms, a waiting space, a phlebotomy/laboratory area, and a bathroom, and electricity is provided by an on-board generator. The Spot offers an array of services including buprenorphine-based MOUD, HIV/STI/HCV testing and treatment on-site, wound care, overdose prevention and response training, and case management. The Spot is staffed by two clinicians (nurse practitioner and MD), a dedicated case manager, and a phlebotomist. The case manager, a licenced social worker, assists clients with various social service needs and facilitates health insurance enrolment for those who are uninsured. Through offering an integrated package of services for PWUD, The Spot aims to normalise the concept of biomedical services to maintain and improve health among this population.

### Study design and participants

This qualitative study was embedded into a cluster-randomized trial designed to determine the community-level effectiveness of an integrated care mobile clinic co-located with a mobile SSP [ClinicalTrials.gov Identifier NCT0356717]. We recruited PWUD from The Spot and SSP vans at four sites in Baltimore, Maryland between March and October 2019. Recruitment site selection (four sites out of six total) was based on the larger study’s recruitment and/or the days The Spot was able to have recruitment occur based on staffing and client flow. Persons were eligible to participate in an interview if they were 18 years or older and a current client at The Spot and/or the SSP. We aimed to interview approximately equal numbers of participants that had and had not used services on The Spot. By interviewing clients and non-clients, we hoped to better understand the range of perceptions of The Spot and barriers to its utilization by PWUD, and identify potential unique barriers faced by those who had not used the services offered. The number of interviews conducted was determined by the scope and nature of the study (e.g. narrow scope) [77], recommendations in the literature [78–80], and data saturation, defined as the point where no new information relevant to the research questions is obtained from additional interviews [81]. All participants provided oral consent prior to participation, and participant consent date and time were documented by the interviewer. Oral consent was obtained due to the stigmatization of drug use and the minimal risk of participation in the interview. Through the consent, participants were informed that the interviews were confidential and findings shared with The Spot and SSP staff would not be linked to any individual participant. The Johns Hopkins University School of Medicine Institutional Review Board (IRB) approved all protocols (IRB-5; IRB00147873).

### Data collection

Eligible individuals were invited to complete the interview in a private room within a nearby research van after receiving their services from The Spot and/or SSP. Interviews were conducted by a medical anthropologist (SG) who was not associated with The Spot or SSP. The interview guide followed a semi-structured format and included discussions about several topics related to healthcare utilization and drug use, including current health concerns and healthcare use, positive and negative healthcare experiences (including experiences of drug use stigma), current drug use behaviours, access to and utilization of harm reduction services, and current and/or previous experiences with MOUD. Clients of The Spot were also asked about their experiences utilizing the mobile clinic, and non-clients were asked about their awareness of The Spot and interest in using an MHC. Demographic information (e.g. age, gender, race/ethnicity) was collected at the start of each interview. The interview guide was initially developed by SG and SS and then reviewed by other members of the team, including healthcare providers on The Spot. Interviews lasted approximately 1 h and were audio-recorded with participant permission. Participants were compensated $20, and water and snacks were available.

### Data analysis

The audio recordings were transcribed verbatim by a professional transcription company and cleaned of identifying information (e.g. names). Data analysis of the text was conducted using an iterative, thematic constant comparison process [82]. The lead qualitative researcher (SG) independently read through six transcripts to develop the initial coding framework via open coding [83]. The initial codebook was developed by collapsing codes from the open coding list based on similarities and differences. Multiple iterations of the codebook were created through coding of additional transcripts and subsequent reflection and discussion by three researchers (SG, SW, SS). Using the constant comparison method, codes were compared within a single interview and between interviews, and variability was considered based on gender and client type (The Spot client vs. non-client). This iterative method facilitated the refinement of existing codes and the identification of emergent codes and illuminated potential relationships between codes [82,84,85]. Two coders (SG, SW) then independently applied the final 84 codes systematically to each of the transcripts in Atlas.ti software; the lead qualitative researcher resolved any discrepancies. When coding was complete, codes were aggregated into thematic categories based on how the codes related to one another. The final thematic framework provides a higher level of contextual framing of the codes and coded text.

## Results

Thirty-one interviews were conducted with PWUD – 16 with persons who were current clients of The Spot and 15 who were current clients of the SSP but had never received services at The Spot. Participant characteristics for each group are reported in Table 1. On average, participants were 46.5 years and roughly half identified as male. Among The Spot clients, the majority identified as non-Hispanic Black (88%), whereas the majority (93%) of non-clients identified as non-Hispanic white. Non-Spot clients were more likely to report being unstably housed (60% and 19%, respectively) and being HCV positive (87% and 19%, respectively) than The Spot clients. All non-clients were current injection drug users. Six (40%) of The Spot clients had a history of injection drug use, but none reported injection drug use in the past month. However, 10 (67%) of The Spot clients did report non-injection drug use in the past month. The majority of participants (84%) reported using MOUD; 67% of non-clients currently used methadone and 100% of The Spot clients currently used buprenorphine. Discussion about The Spot centred on three domains: awareness of The Spot, perceived benefits of The Spot, and barriers to utilization of The Spot.

**Table 1.** Participant characteristics of non-clients (*n* = 15) and clients (*n* = 16) of the healthcare on the spot mobile health clinic in Baltimore, Maryland.

Characteristic	Non-clients	The spot clients	Total
Gender			
Male	7 (47%)	9 (56%)	16 (52%)
Female	8 (53%)	7 (44%)	15 (48%)
Age (mean, SD)	46.7 (8.6)	49.3 (12.3)	46.5 (10.9)
Race/ethnicity			
Non-Hispanic White	14 (93%)	1 (6%)	15 (48%)
Non-Hispanic Black	1 (7%)	14 (88%)	15 (48%)
Hispanic Black	0 (0%)	1 (6%)	1 (3%)
Housing			
Stably housed	3 (20%)	13 (81%)	16 (52%)
Unstably housed	3 (20%)	0 (0%)	3 (10%)
Homeless	9 (60%)	3 (19%)	12 (39%)
Hepatitis C positive	13 (87%)	3 (19%)	16 (52%)
Drug use			
Ever injected drugs	15 (100%)	6 (40%)	21 (68%)
Currently injects drugs	15 (100%)	0 (0%)	15 (48%)
Drug use in past month	15 (100%)	10 (63%)	25 (81%)
Current medication for opioid use disorder			
Methadone	10 (67%)	0 (0%)	10 (32%)
Buprenorphine	0 (0%)	16 (100%)	16 (52%)

### Perceived benefits of the spot

#### Caring, non-judgmental healthcare providers

Participants had often experienced drug-use stigma while accessing healthcare services previously but felt that The Spot provided an opportunity to receive services without such judgement. Since The Spot was co-located with the SSP, non-clients trusted that The Spot staff would treat them the same as the SSP staff do – without any judgement. For example, a 42-year-old female non-client explained: “They’re coming in this neighborhood so they pretty much know what to expect… Yeah, so I’m thinking they know what to expect so they’ll be a little bit more sympathetic”.

Non-clients shared an expectation that The Spot staff would be caring and non-judgmental, and the clients described this as true. When discussing their experiences using services at The Spot, participants overwhelmingly focussed on the van staff: “I think they’re great. I think they help people, they care, they’re nice, I couldn’t imagine it being anything better than that, what they are now. I think they’re doing a good job”. The staff was described as being welcoming, positive, caring, considerate, non-judgemental, encouraging, and supportive. Talking about the support he felt from his provider, a 32-year-old male client discussed being open about drug use:

Participant: Yeah, I tell them [about recent drug use], yeah, I’m always honest, that’s the one thing the lady told me was, “Just be honest with me upfront.” And I have, and I have.

Interviewer*:* How has that been, talking about that?

Participant: Helpful…Because first of all I could feel they know I’m telling the truth and then it’s just like they’re not just giving up on me, you know, they’re working with me, they’re trying to work around, seeing how we could fix it or how we can adjust it to make it work and make it better and I really appreciate that.

Many participants spoke about the providers with great emotion, getting overwhelmed by the support and encouragement they have received on the van. A 25-year-old female client said, crying, “He [the provider] makes me feel like I can do it. You know, I can do it…People are seeing you. You’re trying to get it [stop using drugs]”.

#### Social and physical space

The social and physical space provided by The Spot was viewed as a strength of the MHC among clients, although a few non-clients did acknowledge this, feeling that The Spot allowed for greater privacy:

I’d rather walk out and [go to The Spot] than have to get all the way to the hospital. I would probably avoid that because it’s just a longer, drawn-out process. This is, I feel, more secluded and—you know what I mean? Just more one-on-one, like not—to go in front of a hospital and everybody’s around and stuff. (39-year-old female non-client)

Although small, clients were comfortable receiving services in the mobile clinic. This partially was influenced by the social space created by the staff. Clients spoke, for example, of the positive environment on the van: “[The] people are very kind and they sincerely care, they really do and they’re down to earth. You can just feel the aura, it’s definitely a positive feeling as soon as you step on that van” (32-year-old male client). Similarly, a 53-year-old female client said, “They’re cool. <laughs>… You come in there with your face down and they make you laugh before you leave”. Physically, the space allowed for a confidential conversation, and clients did not have concerns regarding the security of their privacy: “Everybody still has their own personal space [to be seen]” (32-year-old male client). Similarly, a 63-year-old female client felt comfortable, saying, “They [people waiting on the van to be seen] can’t hear you. Well, I can’t hear nobody when they’re in there”. Overall, the social and physical space was viewed as positive: “The madness is out there [outside]. There’s nothing mad about in here. The madness is out there. So once you get on the van you’re pretty much good” (49-year-old male client).

#### Immediate access to treatment

Non-clients and clients believed that an important benefit of The Spot to their communities was that it allowed people to rapidly access treatment when they were ready to do so. A 59-year-old male client said, for example: “Try to keep them out here as long as they can in this area. Because it’s so many people out there lost, man. They lost. Lost…. Sometime what you could have done you might not be able to do later on down the road, so jump at it while it’s there. Things change every day”. Similarly, a 42-year-old female non-client described how much easier it would be for her to access treatment from The Spot when she was ready:

Because, for instance, if I could just walk over there tomorrow, walk in and say, “Listen, I need help, I want to get off the heroin,” and they’re like, “Okay, come on.” Damn! It’s there, instead of me having to find someone’s phone to use, try to call the healthcare provider, make an appointment, wait for the appointment, remember the appointment, get to the appointment, try to survive while waiting for the appointment. That’s a lot. So instant is always better. (42-year-old female non-client)

#### Access to low threshold buprenorphine

Among clients, this access to low threshold buprenorphine was the catalyst for utilizing the MHC and was viewed as beneficial to initiating and continuing their treatment:

You know, a lot of those programs like that—it’s a lot of—you have to—it’s a whole lot of stuff that’s on you, that you’ve gotta do to make sure you stay qualified. I mean, make sure you can still get the medication and stuff like that. And you have very little here. All you’ve got to do is don’t test dirty and you’re good. Show up, get it, and you’re gone. As opposed to some spots you’ve got to go to groups and you’ve got to do this and you’ve got to—you know what I mean? So if it’s anything that’s one of the benefits. It’s like no hassle. Maybe carve out a half hour, 45 minutes of your day once a week, take care of the process, and it’s over. (46-year-old male client)

Some participants had previously been required to attend group sessions as part of their MOUD programs and found this unhelpful – either it did not provide them support and was viewed as a waste of time or talking about drug use increased their desire to use: “By talking about it, talking about it, talking about it that made me want it [drugs] more” (65-year-old male client). Since participants were aware of multiple groups, such as Narcotics Anonymous, they could join if they wanted, most felt that the lack of requirements such as attending a group was beneficial for their success in their MOUD program at The Spot.

#### Accessible healthcare

More broadly, participants believed that people in their communities were benefitted from the ease at which people could access The Spot for healthcare services, generally. A 57-year-old male non-client said, for example, “If it’s something that can help people like me or anybody else, even people that isn’t, or aren’t addicts or whatever, sure. I mean, I think it’s a positive thing. You got so many people out here that are homeless, have nothing…” The Spot was viewed as convenient and as reducing barriers commonly experienced to receiving healthcare services:

We have people [The Spot staff] coming into the community that have the people incoming, that it’s a good thing for people, ‘cause some people don’t have car fare or nothing…but by being right here, I think it’s more convenient, you know what I’m saying, and helpful to the people in the community. (53-year-old female client)

### Barriers to using the spot

#### Lack of awareness

Overall, participants did not feel there were many barriers to receiving healthcare services through The Spot. The most important barrier was simply that the participants did not know it existed: “I had no clue what they did, you know?” A 42-year-old male non-client commented, “I don’t see a reason why nobody wouldn’t want to utilise something that’s free that could help them. I’m going to let people know now that I know because I’m figuring that the people I know don’t know”. This lack of awareness similarly created a barrier to use, initially, among clients. Several clients noted that they made assumptions about the services or were provided wrong information about The Spot. A 46-year-old male explained, for example, “I didn’t know it had nothing to do with drug abuse. I just thought it was a needle exchange. That’s what I thought just by seeing it every day at first”. Another client, a 57-year-old male, was provided inaccurate information: “I had met this guy. He had told me about it…He said as long as you got insurance you was cool”. Importantly, many of the clients were not aware of the additional services provided by the van, thus creating a barrier to utilizing other services: “I really don’t know what all the services that they can provide, you know what I mean?” (59-year-old male).

#### Lack of perceived need

The lack of perceived need was also important, particularly among male non-clients. Clients overwhelmingly learned about The Spot from word of mouth as a place that provided buprenorphine, and this was commonly how non-clients became aware of The Spot as well. As a result of the association of The Spot with buprenorphine, some did not see a need for it. A 57-year-old male non-client, for example, said, “Well, I mean, I just never really, you know, haven’t been sick enough, I don’t think, or been sick to a point where physically, you know, like, an ailment, besides dope sick, you know, needed it”. In addition to the lack of interest in MOUDs generally, for those utilizing methadone, the association of The Spot with buprenorphine also created a lack of perceived need: “I’m on a methadone program, so there would be no need for me to go and get a prescription. It would completely throw me into withdrawal… If I wasn’t on a methadone program, I would definitely go, definitely go to the truck”.

#### Preference for a hospital

Although less common, a few of the male non-client participants felt that they were more likely to seek healthcare at a hospital when needed as this is what they have always done: “I think I’m just so used to going to the hospital that I would probably just go to the hospital”.

#### Prioritization of drug use

Speaking of potential barriers for his peers but not necessarily for himself, a 39-year-old male non-client suggested that people may prioritize their drug use over seeking healthcare services after visiting the SSP: “They’re on the move getting high maybe… they just don’t feel like they have the time”. Prioritization of drug use, however, was not identified by any of the participants as preventing them from accessing healthcare services from The Spot.

## Discussion

This study qualitatively explored the perceived benefits and barriers of utilizing a mobile healthcare clinic by people who use drugs in Baltimore, Maryland through in-depth interviews with clients and non-clients of the mobile clinic. Clients and non-clients perceived The Spot as a benefit to their communities and themselves by improving access to healthcare services, providing access to low-threshold buprenorphine dispensation, and offering services without drug use stigma. Clients described The Spot staff as supporting and encouraging and felt comfortable in the physical clinical space. Our findings show that The Spot has become a trusted source of MOUD with strong peer referral but has not fully capitalized on its ability to provide other needed services for this population, such as wound care and treatment for HCV.

All of the clients interviewed accessed buprenorphine-based MOUD through The Spot, and the low threshold for access was identified by participants as critical to their use of, and success with, their buprenorphine program on The Spot. Despite being safe and effective, buprenorphine continues to be inaccessible and underutilized in the U.S. partially because of barriers to access [41–44,50]. Low barrier programs aim to remove barriers to program utilization, including requiring admissions processes with multiple visits, discontinuation of MOUD for relapse on drug testing, and requiring counselling or participation in 12-step programs [86,87]. Removing these barriers, low-threshold programs generally provide treatment in non-traditional settings, allow people to start treatment the day of entry, utilize a harm reduction approach, and offer flexibility. Providers work with their patients based on their individual needs and desires, offering a non-judgmental approach [55,88]. The Spot clients identified the ease of access, reduced barriers to retention, and lack of drug use stigma from a caring and supportive staff as highly beneficial to themselves as well as to their community. Low barrier MOUD programs have demonstrated feasibility in engaging and retaining marginalized populations in treatment [57,89,90], and emerging data from program adaptation during the COVID-19 pandemic, including reduced toxicology screening and use of telemedicine, reinforce this [91]. Importantly, low barrier MOUD programs may facilitate reduced overdose and all-cause mortality [92,93]. Additionally, buprenorphine treatment disparities have been observed, with treatment concentrated among white persons [94,95]. The Spot’s client base is largely Black [75], suggesting that low barrier buprenorphine access via MHCs may improve equitable access to this life-saving medication.

An important component of low barrier MOUD programs is the patient-provider relationship, which is focussed on individual needs and desires and the lack of judgement towards the patient [55,88]. For patients in underserved and/or stigmatized communities, the patient-provider relationship has a critical impact for engagement in care, retention in care, and health outcomes, including for people with low-income, people who are homeless [96], people living with HIV [97,98], and people who inject drugs [99]. Research suggests that many MHCs demonstrate an ability to foster trusting relationships between provider and patient [64,65,70,100,101]. Qualitative research exploring experiences with MHC demonstrates that people value the ways in which the providers make people feel welcomed, are easy to talk to, engage without judgement, and understand their community [65,70,100]. In a survey of homeless persons who use drugs attending an MHC in Boston, Massachusetts, Fine and colleagues found that 98.8% of respondents trusted and felt respected by the program staff [101]. Through the descriptions of client experiences on The Spot, our findings further demonstrate the ability of MHC, and importantly those serving PWUD, to promote trusting and respectful provider-patient relationships. PWUD often encounter interpersonal barriers (e.g. stigma from healthcare providers) in traditional health settings, which negatively impact their engagement in care and, ultimately, the health and wellbeing of PWUD [18,19,22,102]. By addressing this barrier as well as others (e.g. difficulty navigating the healthcare system, lack of insurance, burden of appointment), MHC may have an important role in addressing health disparities experienced among this population.

Despite the achievements of The Spot van, the positive feedback from clients, and its co-location with a mobile syringe service program van, barriers exist with respect to awareness of the MHC, generally, and the specific services provided. Clients and non-clients aware of The Spot overwhelmingly learned about The Spot through word of mouth. Although The Spot offers a range of services, clients typically learned about The Spot through social contacts who informed them of its buprenorphine dispensation, and many referred to The Spot as the “bup van”. As a result, PWUD who are not interested in receiving buprenorphine, and especially those in methadone programs, may be excluded from peer communications about The Spot van. Previous research has documented the importance of peer-to-peer information sharing among PWUD, however, which has been linked to healthcare utilization within populations of PWUD [103–105]. In addition to sharing flyers or pamphlets with other service organizations for PWUD and having greater information dissemination by the SSP staff, awareness of The Spot and its services could be aided through the use of formalized peer dissemination and recruitment strategies that ensure diverse messages about The Spot services [106–108]. This study provides further support for the role of MHC in providing healthcare services to vulnerable populations, generally, and people with OUD specifically. This research, though, is not without limitations. This study recruited MHC clients from one mobile clinic in Baltimore, Maryland. It is possible that the perceptions and experiences of MHC use by clients and non-clients could vary geographically and/or by the specifics of the MHC. Although client and non-client participants were recruited from each of the four recruitment locations, the racial distribution of clients and non-clients differed greatly. This is not unexpected, as 77% of The Spot clients were Black at the time of data collection [75], and up to 70% of SSP clients are white at some locations (unpublished data). However, as a result, we were unable to explore possible differences in perceptions and utilization of The Spot based on race and ethnicity. The Spot clients were introduced to the study by their provider in The Spot, and non-clients were approached by study staff outside of the SSP; participants were interviewed immediately after their clinic or SSP visit. While participants were informed that their providers and/or SSP staff would not have access to the specific information shared in the interview and that what they shared would not impact their current or future service at The Spot or SSP, it is possible that participants may have felt uncomfortable providing criticisms or critiques. Only current clients of The Spot were interviewed. It is possible that individuals who received services at The Spot at one time and then stopped may have different perspectives of The Spot and their experience. Finally, interviews occurred prior to the COVID-19 pandemic and the loosening of regulations on buprenorphine and methadone prescribing.

Mobile health clinics offering low-threshold services, such as The Spot, provide an opportunity to increase healthcare accessibility and reduce health disparities among vulnerable populations [59,68]. The Spot was implemented to work in partnership with the city health department’s SSP to provide healthcare services to people who use drugs in impoverished and underserved Baltimore neighbourhoods, with a particular focus on addiction, HCV, and HIV treatment. Low-threshold buprenorphine dispensation and service provision without drug use stigma were identified by clients as strengths of The Spot. Innovative strategies to inform people who use drugs about the range of services offered by The Spot, however, are greatly needed. These findings contribute to the literature on the role of MHC in providing healthcare services to vulnerable populations, generally, and people with OUD in particular.

## Data Availability

The data that support the findings of this study are available on request from the corresponding author, SMG. The data are not publicly available due to their containing information that could compromise the privacy of research participants.
